# CSACI position statement: transition recommendations on existing epinephrine autoinjectors

**DOI:** 10.1186/s13223-021-00634-2

**Published:** 2021-12-13

**Authors:** Lucy Dong Xuan Li, Elissa M. Abrams, Elana Lavine, Kyla Hildebrand, Douglas Paul Mack

**Affiliations:** 1 Department of Paediatrics, Division of Clinical Immunology and Allergy, University of Toronto, Toronto, ON Canada; 2 Department of Paediatrics, Section of Allergy and Clinical Immunology, University of Manitoba, Winnipeg, MB Canada; 3 Department of Paediatrics, University of Toronto, Toronto, ON Canada; 4 Department of Paediatrics, Queen’s University, Kingston, ON Canada; 5 Department of Paediatrics, Division of Allergy and Immunology, University of British Columbia, Vancouver, BC Canada; 6 Department of Paediatrics, Paediatric Allergy, Asthma, and Immunology, McMaster University, Hamilton, ON Canada; 7 Halton Pediatric Allergy, Burlington, ON Canada

**Keywords:** Epinephrine, Anaphylaxis, CSACI position statement, 0.5 mg epinephrine autoinjector

## Abstract

Epinephrine is the first line treatment for anaphylaxis, an acute potentially life-threatening allergic reaction. It is typically administered intramuscularly in the anterolateral thigh at a dose of 0.01 mg/kg of 1:1000 (1 mg/ml) solution to a maximum initial dose of 0.5 mg. Currently in Canada, epinephrine autoinjectors (EAI) are available in three doses, 0.15 mg, 0.30 mg, and 0.50 mg. There are currently no published studies comparing 0.3 mg and 0.5 mg EAIs in the paediatric or adult populations to compare clinical effectiveness. However, as weight increases above 30 kg, the percentage of the recommended 0.01 mg/kg epinephrine dose from an existing 0.3 mg EAI decreases resulting in potential underdosing. As such, The Canadian Society of Allergy and Immunology (CSACI) recommends that for those who weigh ≥ 45 kg, physicians could consider prescribing the 0.50 mg EAI based on shared decision making with patients.

## Background

### Anaphylaxis and the importance of epinephrine

Anaphylaxis is an acute potentially life-threatening allergic reaction with a lifetime prevalence between 1.6 and 5.1% [1]. In children, food allergy is a leading cause of anaphylaxis, while medications and insect venom are more common causes in the adult population [2]. While definitions vary, anaphylaxis is a clinical diagnosis and is characterized as a severe reaction that may involve a wide range of organ systems and is typically rapid in onset. The World Allergy Organization (WAO) Anaphylaxis Committee in 2020 modified the existing National Institute of Allergy and Infectious Diseases and the Food Allergy and Anaphylaxis Network (NIAID/FAAN) criteria with an aim to simplify the criteria by combining the first two NIAID/FAAN criteria and modifying the third (Table 1) [3, 4]. While anaphylaxis may be life-threatening, fatal anaphylaxis is a rare occurrence and has been estimated to range from 0.064 to 0.099 deaths per 100,000 population per annum in the UK, US, and Australia [5].

**Table 1:** Clinical criteria for diagnosing anaphylaxis

WAO 2020 Criteria [3]: anaphylaxis is likely when any one of the following 2 criteria are fulfilled:	NIAID/FAAN Criteria [4]: fulfilling any 1 of the following 3 criteria indicates anaphylaxis is highly likely
Criterion 1:	Criterion 1:
Acute onset of an illness (minutes to several hours) with simultaneous involvement of the skin, mucosal tissue, or both (ex: generalized hives, pruritus or flushing, swollen lips-tongue-uvula) and at least one of the following:	Sudden onset of an illness (minutes to several hours), with involvement of the skin, mucosal tissue, or both (ex: generalized hives, itching or flushing, swollen lips-tongue-uvula) and at least one of the following:
(i) Respiratory compromise (ex: dyspnea, wheeze-bronchospasm, stridor, reduced peak expiratory flow, hypoxemia)	(i) Sudden respiratory symptoms and signs (ex: shortness of breath, wheeze, cough, stridor, hypoxemia)
(ii) Reduced BP or associated symptoms of end-organ dysfunction (ex: hypotonia, syncope, incontinence)	(ii) Sudden reduced BP or symptoms of end-organ dysfunction (ex: hypotonia, incontinence)
(iii) Severe gastrointestinal symptoms (ex: severe crampy abdominal pain, repetitive vomiting), especially after exposure to non-food allergens	Criterion 2:
Criterion 2:	Two or more of the following that occur suddenly after exposure to a *likely allergen or other trigger for that patient* (*minutes to hours*)
Acute onset of hypotension or bronchospasm or laryngeal involvement after exposure to a known or highly probably allergen for that patient (minutes to several hours), even in the absence of typical skin involvement	(i) Sudden skin or mucosal symptoms and signs (ex: generalized hives, itch-flush, swollen lips-tongue-uvula)
	(ii) Sudden respiratory symptoms and signs (ex: shortness of breath, wheeze, cough, stridor, hypoxemia)
	(iii) Sudden reduced BP or symptoms of end-organ dysfunction (ex: hypotonia, incontinence)
	Sudden gastrointestinal symptoms (ex: cramping abdominal pain, vomiting)
	Criterion 3:
	Reduced blood pressure (BP) after exposure to a *known allergen for that patient* (*minutes to hours*)
	(i) Infants and children: low systolic BP (age specific) or greater than 30% decrease in systolic BP
	(ii) Adults: systolic BP of less than 90 mm Hg or greater than 30% decrease from that person’s baseline

The first line treatment for anaphylaxis is epinephrine which is typically administered intramuscularly (IM) in the anterolateral thigh at a dose of 0.01 mg/kg of 1:1000 (1 mg/ml) solution to a maximum initial dose of 0.5 mg [2]. The dose recommendations for anaphylaxis have been based on clinical experience and extrapolated from uses of epinephrine in other conditions and limited data exist to determine the precise dose for all patients. Epinephrine functions via its effects on alpha-1-adrenergic, beta-1-adrenergic, and beta-2-adrenergic receptors to decrease airway edema, increase peripheral vascular resistance via vasoconstriction, induce cardiac inotropic and chronotropic effects, stimulate bronchodilation, and decrease mediator release [2].

While syringe and ampule administration may be reasonable in hospitals and clinics, patients and/or caregivers do not typically have access to these supplies and this method may increase risks of inaccurate dosing and delays in administration, even amongst health care professionals [6]. Until recently in Canada, epinephrine autoinjectors (EAI) were only available in fixed doses of 0.15 mg and 0.3 mg. Product monographs for such autoinjectors advise the usage of 0.15 mg autoinjectors for individuals weighing 15–30 kg and the 0.3 mg autoinjectors for those weighing ≥ 30 kg. However, these doses potentially result in suboptimal dosing in patients not weighing exactly 15 or 30 kg.

Currently, various medical professional organizations recommend that appropriate dosing of EAIs based on weight should allow for flexibility at the highest and lowest weights in the ranges suggested by product monographs. The CSACI has previously published a guideline suggesting that infants < 15 kg should be prescribed the 0.15 mg dose of autoinjector, despite its published monograph [7]. Similarly, other recommendations have suggested that patients weighing < 25 kg should use the 0.15 mg dose and those ≥ 25 kg should switch to the 0.3 mg dose [7, 8]. In fact, Australian guidelines suggest that this dose transition occur even lower, at 20 kg [9]. In October 2020, an EAI became available in Canada for the first time with a dose of 0.5 mg. Although the product monograph for that device advises the 0.5 mg dose for individuals over 60 kg, no guidelines to date have outlined when a prescriber should consider transitioning to the 0.5 mg dose in the paediatric population. It has been suggested that a 40 kg individual would receive only 75% of the ideal dose with a 0.3 mg EAI, using the weight-based per kilogram dose indicated above [10].

There are currently no published studies comparing 0.3 mg and 0.5 mg EAIs in the paediatric or adult populations to compare clinical effectiveness. Pharmacokinetically, the plasma concentration of epinephrine peaks at 5 min and 30–50 min after injection [11, 12]. Turner et al. demonstrated that increasing the injected dose from 0.3 mg to 0.5 mg while using an adequate needle length will increase the plasma level of epinephrine [13]. The dose recommendation for treatment of anaphylaxis with 0.01 mg/kg (maximum 0.5 mg) of 1 mg/ml epinephrine solution given IM has been based on clinical experience but has also been extrapolated from literature supporting the doses of epinephrine used in other conditions.

Needle length of the various EAIs have also been studied and appears to be a potential concern when administering the 0.15 mg autoinjectors into patients weighing less than 15 kg. Studies have shown that in infants who are < 15 kg, the needle length could potentially hit the bone in 29% to 43% of cases [14, 15]. In adults, the anterolateral aspect of the thigh is higher in women than men and as such, the 0.3 mg device may not reach the muscle in many women [16–18]. With this in mind, the new 0.5 mg EAI available in Canada has a needle length of 23 mm which is longer than the existing 0.15 mg and 0.30 mg EAIs available.

Clinically, it has been suggested that individuals who have a larger body habitus should carry the 0.5 mg epinephrine autoinjector. However, to date, there have been no studies comparing epinephrine levels after epinephrine autoinjector use by obese vs non obese patients. In one study involving 261 children and 60 adults with anaphylaxis, 22% subjects with obesity did not need repeat dosing of epinephrine more than non-obese patients suggesting that pre-existing epinephrine autoinjectors can work well in obese patients [19].

This position statement will address a number of questions regarding epinephrine prescribing and administration for the paediatric population at risk of anaphylaxis, based on the EAIs currently available in Canada.

### What products are available?

Currently in Canada, there are three doses for EAIs—0.15 mg, 0.30 mg, and 0.50 mg (Table 2).

**Table 2:** Available epinephrine autoinjectors in Canada with current product monograph at time of publication and CSACI recommendations

Brand	Available doses (mg)	Product monograph recommendations	CSACI recommendations
EpiPen^®^	0.30	For those who weigh ≥ 30 kg	EpiPen® is recommended for those ≥ 25 kg
EpiPen Jr^®^	0.15	For those who weigh between 15 and 30 kg	For those < 25 kg, the EpiPen Jr® is recommended
Allerject^®^	0.30	For those who weigh ≥ 30 kg	For those ≥ 25 kg, the Allerject® 0.30 mg is recommended
Allerject^®^	0.15	For those who weigh 15–30 kg	For those < 25 kg, the Allerject® 0.15 mg is recommended
Emerade™	0.50	Adult: > 60 kg: 0.3 to 0.5 mg depending on clinical judgement	For those ≥ 45 kg, the Emerade™ 0.5 mg is recommended
Emerade™	0.30	Pediatric: Children > 30 kg: 0.3 mg	For those ≥ 25 kg to < 45 kg, the Emerade™ 0.30 mg is recommended
		Adolescent > 30 kg: The dosage recommendations for adult patients should be followed	
		Adult: < 60 kg: 0.3 mg	

Given the lack of other available EAI doses, product monographs for the existing EAIs are conservative in that, at certain transition weights, significant underdosing could theoretically occur when compared to the weight-based per kilogram doses indicated above.

Existing guidelines and position statements have addressed the issues concerning limited pre-fixed EAI doses and dose transitions.

### Are there risks associated with underdosing epinephrine?

Despite the current, internationally accepted recommendation of administering 0.01 mg/kg of epinephrine to treat anaphylaxis, it is presently impossible to administer that exact dose to a child weighing between 15 and 30 kg with existing 0.15 and 0.3 mg EAIs. For example, an average 7 year old child who weighs 22.5 kg, administered an EAI of 0.15 mg, receives a 33% underdose; administering an EAI of 0.3 mg delivers a potential 33% overdose [20]. Similarly, using a 0.3 mg EAI for a 50 kg adult would result in a 40% underdose. As such, physicians face a common dilemma of choosing whether to potentially under- or over- dose a patient with existing EAIs.

From a safety standpoint, risks of underdosing include inadequate treatment of anaphylaxis, further progression of an evolving allergic/anaphylactic reaction, and a potential increase in the risk of a biphasic reaction [21–23]. In fact, individuals who have biphasic reactions require more epinephrine than those who experience a single phase reaction In one study of 134 patients with anaphylaxis for which data was available for 103 patients, 19.4% of these individuals had biphasic reactions. Biphasic reactors were administered less epinephrine (p = 0.048) and also took significantly longer to achieve resolution of their initial symptoms compared to those who experienced a single phase reaction [23]. In addition, a large Canadian study demonstrated that the risk of uncontrolled reactions (defined as requiring 2 or more doses of epinephrine) was nearly five times higher amongst children who did not receive epinephrine in the prehospital setting [24].

### Are there risks associated with overdosing epinephrine?

The risk of epinephrine overdose predominantly occurs with the administration of intravenous epinephrine [25–30]. Errors in weight-based calculations, epinephrine concentrations, and overly rapid rate of administration all contribute to adverse events observed with IV epinephrine administration. A higher risk of cardiovascular complications such as arrhythmia, ischemia, stroke, hypertension, coronary dissection and infarction have been reported with intravenous bolus epinephrine [27]. Case reports in paediatrics have reported loss of consciousness and severe pulmonary edema with accidental overdose of intravenous epinephrine [25–30].

In the adult population, a rise in troponin levels have been seen predominantly after administration of intravenous bolus epinephrine [31]. Patients with pre-existing cardiovascular disease who take medications such as beta-blockers may be at higher risk of developing severe anaphylaxis. In such patients who present in acute anaphylaxis, the decision of whether to administer epinephrine and how much may pose as a therapeutic dilemma. However, the presence of cardiovascular disease does not forbid the use of epinephrine in anaphylaxis as no other medications have the life saving qualities of epinephrine in this medical emergency.

Until a wider variety of fixed doses of EAI devices becomes available, risks of excessive epinephrine doses will continue, however typically these risks are mild with intramuscular injection and are balanced by the risk of morbidity associated with lack of use. In a study of administration of 0.15 mg via EAI versus the 0.3 mg EAI in children between the ages of 4–8 years old and who weighed 15–30 kg, and were at risk for anaphylaxis, children who received 0.3 mg of IM epinephrine (n = 5) were found to have a higher systolic blood pressure as well as mean blood glucose compared to those who received 0.15 mg of IM epinephrine [11]. All children experienced transient expected symptoms such as pallor, tremor, and anxiety. Those who had received EpiPen 0.3 mg also developed palpitations, headache, and nausea, with 1 child experiencing QTc prolongation [11]. Similar transient adverse events were observed in an adult cohort of men between the ages of 18–35 years who received 0.3 mg of intramuscular epinephrine [12].

### What are the current recommendations for EAI dosing?

Currently, the CSACI advises that an EAI of 0.15 mg be prescribed for children weighing less than 15 kg [7]. Similar recommendations have been made by other societies such as the WAO that advises the usage of 0.15 mg EAI in children between the ages of 1–5 years [3], ASCIA advising 0.15 mg for children weighing 7.5–20 kg [9], EAACI advising 0.15 mg for those weighing between 7.5 and 25 kg [32], and the AAAAI advising 0.15 mg for those individuals weighing between 10 and 25 kg [33]. In the context of vaccine anaphylaxis, the Canadian Immunization Guide has advised that a 0.15 mg EAI be used for those weighing up to 20 kg, a 0.3 mg EAI be used for those weighing between 21 and 45 kg and a 0.5 mg EAI be used for those ≥ 46 kg [34].

For higher fixed doses of the epinephrine autoinjector, the WAO advises that 0.3 mg should be used for children aged 6–12 years and that 0.5 mg be used for teenagers and adults [3]. Previous Canadian consensus, EAACI, and AAAAI all advise that those who weigh ≥ 25 kg carry a 0.3 mg EAI [32, 33, 35]. ASCIA and the Canadian Immunization guide outlines the use of 0.3 mg for children and adults who are ≥ 20 kg indicating professional acceptance of a significantly higher proportional dose of epinephrine than 0.01 mg/kg [9, 34].

### What does the CSACI advise on the 0.5 mg EAI?

The universally accepted and guideline-recommended epinephrine dose of 0.01 mg/kg itself is based on clinical experience and the ideal dose hasn’t been well studied in most patient populations. Thus, discussions on optimal doses in general populations rely largely on limited evidence and professional consensus and guidelines. As weight increases above 30 kg, the percentage of the recommended 0.01 mg/kg epinephrine dose from an existing 0.3 mg EAI decreases resulting in potential underdosing. While it is reasonable that the dose transition can occur at 45 kg, there are a number of key considerations.

#### Can an EAI of 0.5 mg be safely prescribed to patients weighing ≥ 45 kg?

While no clear data has evaluated the safety of 0.5 mg epinephrine at this weight, extrapolations from other studies suggests that this 11% overdose is unlikely to be harmful. The CSACI recommends further study of the optimal dose.

#### Is a dose of 0.5 mg superior to 0.3 mg in patients weighing ≥ 45 kg?

Head to head clinical trials comparing 0.3 mg and 0.5 mg for the treatment of anaphylaxis have not been published. However, since the universally recommended dose is 0.01 mg/kg, it is reasonable to prescribe the 0.5 mg dose in patients weighing ≥ 45 kg. Since there is currently only one device offering this dose, the decision to use the 0.5 mg dose may be influenced by patient preferences regarding device design and should be decided through shared-decision making. Considerations for co-morbid risk factors such as asthma, β-blocker use, body habitus, pregnancy, cardiac disease and prior severe anaphylaxis may also influence this decision. The CSACI recommends further study of the optimal treatment dose.

## Conclusion

In addition to pre-existing guidelines on the transition of epinephrine autoinjector dose for children < 15 kg, ≥ 15 to < 25 kg, and ≥ 25 kg, we suggest that a 0.5 mg EAI may be considered to those who are ≥ 45 kg. Epinephrine is the first line treatment in anaphylaxis and adverse events related to a higher dose of epinephrine appear to be mild and transient in both children and adults. Through shared-decision making, some patients and clinicians may decide to use a lower dose based on patient preference for specific EAIs.

## Data Availability

Not applicable.

## Abbreviations

EAI: Epinephrine autoinjector
CSACI: Canadian Society of Allergy and Immunology
WAO: World Allergy Organization
NIAID: National Institute of Allergy and Infectious Diseases
FAAN: Food Allergy and Anaphylaxis Network
BP: Blood pressure
mg: Milligram
kg: Kilogram
IM: Intramuscular
IV: Intravenous
ASCIA: Australasian Society of Clinical Immunology and Allergy
EAACI: European Academy of Allergy and Clinical Immunology
AAAAI: American Academy of Allergy, Asthma, and Immunology
